# The effect of electronic monitoring feedback on medication adherence and clinical outcomes: A systematic review

**DOI:** 10.1371/journal.pone.0185453

**Published:** 2017-10-09

**Authors:** Milou van Heuckelum, Cornelia H. M. van den Ende, Anne E. J. Houterman, Charlotte P. M. Heemskerk, Sandra van Dulmen, Bart J. F. van den Bemt

**Affiliations:** 1 Departments of Rheumatology and Pharmacy, Sint Maartenskliniek, Nijmegen, The Netherlands; 2 Department of Rheumatology, Radboud University Medical Center, Nijmegen, The Netherlands; 3 Department of Pharmacy, Radboud University Medical Center, Nijmegen, The Netherlands; 4 Department of Pharmaceutical Sciences, University of Utrecht, Utrecht, The Netherlands; 5 NIVEL (Netherlands Institute for Health Services Research), Utrecht, The Netherlands; 6 Faculty of Health Sciences, Buskerud and Vestfold University College, Drammen, Norway; 7 Department of Primary and Community Care, Radboud University Medical Center, Nijmegen, The Netherlands; Iowa State University, UNITED STATES

## Abstract

**Objective:**

This study aims to assess the efficacy of Electronic Monitoring Feedback (EMF) as an intervention to improve medication adherence (i.e. dose- or full adherence) and clinical outcomes in adult patients.

**Methods:**

A systematic search was performed in Medline, EMBASE, PsycINFO and Web of Science and reported according to the PRISMA guidelines. Randomised controlled trials (RCTs) comparing EMF with usual care were identified to systematically summarise the evidence for use of EMF in improving medication adherence and clinical outcomes. The GRADE approach was used to assess the quality of the body of evidence.

**Results:**

Of 9,993 initially-identified studies, ten studies (four of high-quality and six of low-quality) were included. The sample size of the studies included varied from 18 to 205 patients. Four of the six studies (66.7%) reported a significant positive effect of EMF on mean dose adherence levels, whereas a significant positive effect of EMF on mean full adherence levels was found in all of the included studies (100%, five out of five of the studies included). A significant positive effect of EMF on clinical outcomes was reported in one of the seven studies included. The overall effect of EMF on mean dose- and full adherence was positive and the overall effect of EMF on clinical outcomes was inconclusive.

**Conclusion:**

Considering the positive effect of EMF on medication adherence, EMF might be a promising intervention to enhance medication adherence. However, the effect of EMF on clinical outcomes was inconclusive. Prior to implementing EMF in clinical practice, future research with high-quality studies (e.g. adequate sample sizes, follow-up periods and no interfering co-interventions) is required to examine the (long-term) efficacy of EMF.

## Introduction

Since drugs can only provide effect when actually taken, non-adherence to medication might worsen clinical outcomes, decrease quality of life, and will eventually lead to increased healthcare costs [1,2]. It is estimated that approximately 50% of all patients who are taking medication for chronic diseases do not adhere to their prescribed medication [3,4]. For five different diseases (hypertension, chronic obstructive pulmonary disease, chronic back pain, depression and rheumatoid arthritis), it has been estimated that productivity loss due to non-adherence to medication leads to annual costs of €28 to €50 billion in the United Kingdom, €38 to €75 billion in Germany and €9 to €13 billion in the Netherlands [2]. Therefore, interventions to enhance medication adherence in clinical practice are urgently needed.

Medication adherence is defined as the extent to which a patient’s medication-intake behaviour corresponds with the medication regime prescribed by the healthcare professional [1,4,5]. Medication-taking behaviour should however not be understood as a single decisional process, but as a three-phase process: the initiation phase (patients must initiate treatment), the implementation phase (patients must implement the right dosing regimen) and the continuation phase (patients must persist with the treatment) [6]. In this review, we will focus on medication adherence. Medication adherence can be expressed in dose- and full adherence. Dose adherence is defined as the ratio between the number of doses taken and the number of doses prescribed, whereas full adherence is defined as dose adherence in the correct time schedule [7]. Several reasons for non-adherence to medication might exist in each of these three phases.

A way to identify non-adherent patients is to directly monitor a patient’s adherence. Electronic devices, such as the Medication Event Monitoring System (MEMS), have been used for several years to measure medication adherence and are seen as a gold standard method to assess patient’s medication-taking behaviour [8]. MEMS have the ability to register both dose- and full adherence, whereas other methods, including questionnaires and refill adherence data, only provide data on dose adherence. The MEMS is an electronic device that consists of a MEMS cap with an electronic circuit that records opening of the MEMS bottle [3,7]. The electronically registered opening intervals offer healthcare professionals insight into the medication-taking behaviour of their patients.

Alongside this, the results from medication-intake behaviour recorded by electronic devices might also be used as an intervention to confront patients with detrimental medication-taking behaviour in particular. Data obtained from these devices provide the opportunity to give patients personalised feedback about their medication-taking behaviour and to intervene in cases of non-adherent behaviour. In cases of adherent behaviour, this technique also provides the opportunity to encourage patients to continue this behaviour [1,7,9]. Such personalised feedback is also known as Electronic Monitoring Feedback (EMF) [7]. In this study, EMF will be classified as instant EMF or non-instant EMF. The latter refers to EMF given by a healthcare professional or researcher after a certain period of use. This type of feedback can be used to address both intentional and unintentional non-adherent behaviour. A disadvantage of non-instant feedback is the evaluation of medication-intake behaviour over a longer time frame, which makes this type of feedback less appropriate for immediate intervention in cases of non-adherent behaviour.

In contrast to non-instant feedback, instant feedback allows to immediate intervention if patients are not taking their medication. MEMS devices with a MEMS-view-cap are considered to provide such instant feedback. A MEMS-view-cap shows the number of MEMS bottle openings over the past 24 hours and enables patients to visualize the number of doses taken. However, this type of feedback addresses only unintentional non-adherent behaviour, whereas intentional non-adherent behaviour is disregarded. As a comprehensive definition for EMF is lacking, we suggest the following: EMF is any personalised feedback (instant or non-instant) on previously electronically obtained adherence data to improve (intentional and/or unintentional non-adherent behaviour), or sustain medication adherence.

Although individual studies suggest a positive effect of EMF on medication adherence and clinical outcomes, results from these studies have not yet been systematically summarised. Therefore, this study aims to systematically summarise the evidence obtained in randomised controlled trials for the efficacy of EMF to enhance medication adherence and clinical outcomes in adult patients.

## Methods

This systematic review, which assess the efficacy of EMF on medication adherence and clinical outcomes, follows the PRISMA-guidelines (S1 Checklist) [10,11].

### Eligibility criteria

Eligible studies were: (1) randomised controlled trials; (2) aimed at enhancing medication adherence and, if assessed, clinical outcomes by EMF (3) in adult patients (aged 18 years or older) compared to usual care. Additional inclusion criteria for these RCTs were:

1. Only studies with EMF as the primary intervention component were included. Co-interventions were only permitted if these co-interventions incorporated a form of personalised feedback that was based on electronically obtained adherence data (for instance, a non-judgmental discussion of the results, identifying barriers and facilitators for (non-)adherent behaviour, problem detection, problem solving techniques, clarifying treatment regime etc.). Instant or direct feedback from monitoring devices itself was also considered as personalised feedback. Studies in which EMF was used to assess the efficacy of an intervention on medication adherence were excluded. Examples of studies that were excluded based on this latter criterion were: Apter et al. (primary intervention component was a problem-solving technique); Wu et al. (pre-recorded time message or programmable medication reminders as primary intervention components); Interian et al. (motivational enhancement therapy for antidepressants); and Wu et al. 2012 (teaching and counselling as primary intervention components) [12–15].
2. Patients in the control group received entirely usual care (the use of electronic devices to measure adherence levels in the usual care group was permitted).
3. Medication adherence (i.e. dose- or full adherence) was the primary outcome measure and clinical outcomes were the secondary outcome measures.

Articles without an abstract and/or access to the full text manuscript were excluded. Studies that focused on institutional care were also excluded. There were no restrictions on language, sample size, diseases/conditions or duration of the follow-up period and articles were not excluded based on missing data regarding primary- and secondary outcomes. So, we focused on both chronic- and acute medication.

### Search strategy

A literature search for this systematic review was performed up until August 8^th^, 2017, in PubMed database, PsycINFO, EMBASE and Web of Science. A detailed search strategy can be found in S1 Appendix. Reference lists of included studies were checked to ensure literature saturation.

### Study selection

Three reviewers (AH, BB and MH) independently selected eligible studies based on their titles and abstracts. Subsequently, full text evaluation of all the potentially eligible studies was independently carried out by the same reviewers (AH, BB and MH). Any disagreements about the final study selection were discussed to achieve consensus.

### Data collection process

Relevant data available from eligible studies were collected in a data-sheet. The following data were extracted:

- Study characteristics: author names, title, date of publication, journal, country in which the study was performed, study design, sample size, study duration and patient characteristics (gender, age, disease and prescribed medication).
- Intervention characteristics: type of intervention, type of (electronic) device, duration of the (pre)intervention period, duration and frequency of feedback, study medication, medication use and feedback provider.
- Outcome measures: medication adherence (i.e. dose- and full adherence) measured with an electronic device (including mean, standard deviation and 95% confidence interval) and data related to clinical outcomes.

Data extraction was conducted by CH; uncertainties were resolved by AH. All outcome data were verified by BB. P-values ≤0.05 were considered as statistically significant. In case of missing information regarding dose- and full adherence, authors were contacted to request the missing data for these primary outcomes.

### Outcome measures and analysis

Medication adherence at the end of the intervention period registered with an electronic device was defined as the primary outcome measure, whereas clinical outcomes were defined as secondary outcomes in this systematic review. The effect of EMF on medication adherence per study was considered positive (+) if a significant effect was found on dose- or full adherence. In case no significant effect of EMF on medication adherence was found, the overall effect was considered negative (-). If studies did not report levels of significance or if results were contradictive, the overall effect per study was assessed as not available (NA) or neutral (+/-), respectively. The effect of EMF on clinical outcomes per study was considered positive (+) when a significant effect was found on at least 66.7% of the reported clinical outcomes in that individual study. For that purpose, we used results on clinical outcomes at the end of the intervention period or the first results after the intervention had been stopped. Otherwise, individual studies were scored as negative (-) or not available (NA) when clinical outcomes or levels of significance were not assessed. Finally, the overall effect of EMF on medication adherence and clinical outcomes was defined as positive if at least 66.7% of the total included studies reported positive results on medication adherence or clinical outcomes. Consensus of these percentage cut-offs was achieved in a consensus meeting (BB, EE and MH). Clinical heterogeneity was assessed by examining types of participants, interventions and outcomes in each study. Two reviewers (BB and MH) assessed clinical heterogeneity during a consensus meeting.

### Quality of evidence

Apart from the data extraction a quality assessment was performed. The criteria for systematic reviews of the Cochrane Collaboration Back Review Group and the criteria described by Hayden et al. were used to assess the risk of bias of the included studies [16,17]. All included studies were independently evaluated by three reviewers (AH, BB and MH). These three individual quality assessments were discussed until consensus was reached. In order to obtain a final quality score, criteria were classified according to critical and less critical criteria during a consensus meeting (attendees: AH, BB and MH). This consensus meeting resulted in a quality assessment model that distinguishes between criteria that are critical and less critical to assess the risk of bias in studies focused on the efficacy of EMF on medication adherence and clinical outcomes. For instance, criteria focused on blinding of patients and blinding of care providers were less appropriate to assess as a risk of bias, due to the inherent awareness of being monitored with the EMF-devices by patients and care providers. The quality assessment model is provided in S1 Table. Studies that scored both ≥ 75% positive on the critical criteria and ≥ 50% positive on the less critical criteria were classified as high-quality studies. These percentage cut-offs were discussed and agreed during a consensus meeting.

The GRADE (Grading of Recommendations Assessment, Development and Evaluation) approach was used to provide an overall grade for the quality of the body of evidence on two predefined patient outcomes: (1). medication adherence and (2). clinical outcomes [18–24]. Although the initial quality of evidence of randomised controlled trials is high, five explicit criteria of the GRADE approach might downgrade the total body of evidence for each patient outcome: risk of bias, inconsistency, indirectness, imprecision, and publication bias [18–24]. Factors that might increase the quality level of evidence are a large magnitude of the studied effect, a dose-response gradient, or the fact that all plausible confounding would reduce the demonstrated effect or suggest a spurious effect when results show no effect [19]. See S2 Table. for a detailed description of methods used to assess the quality of evidence based on these criteria. The GRADE approach was only used for the primary outcome measures. Eventually the body of evidence for each outcome was categorised in one of the specified categories: high, moderate, low, or very low [18–24].

## Results

### Study selection

**Fig 1:**
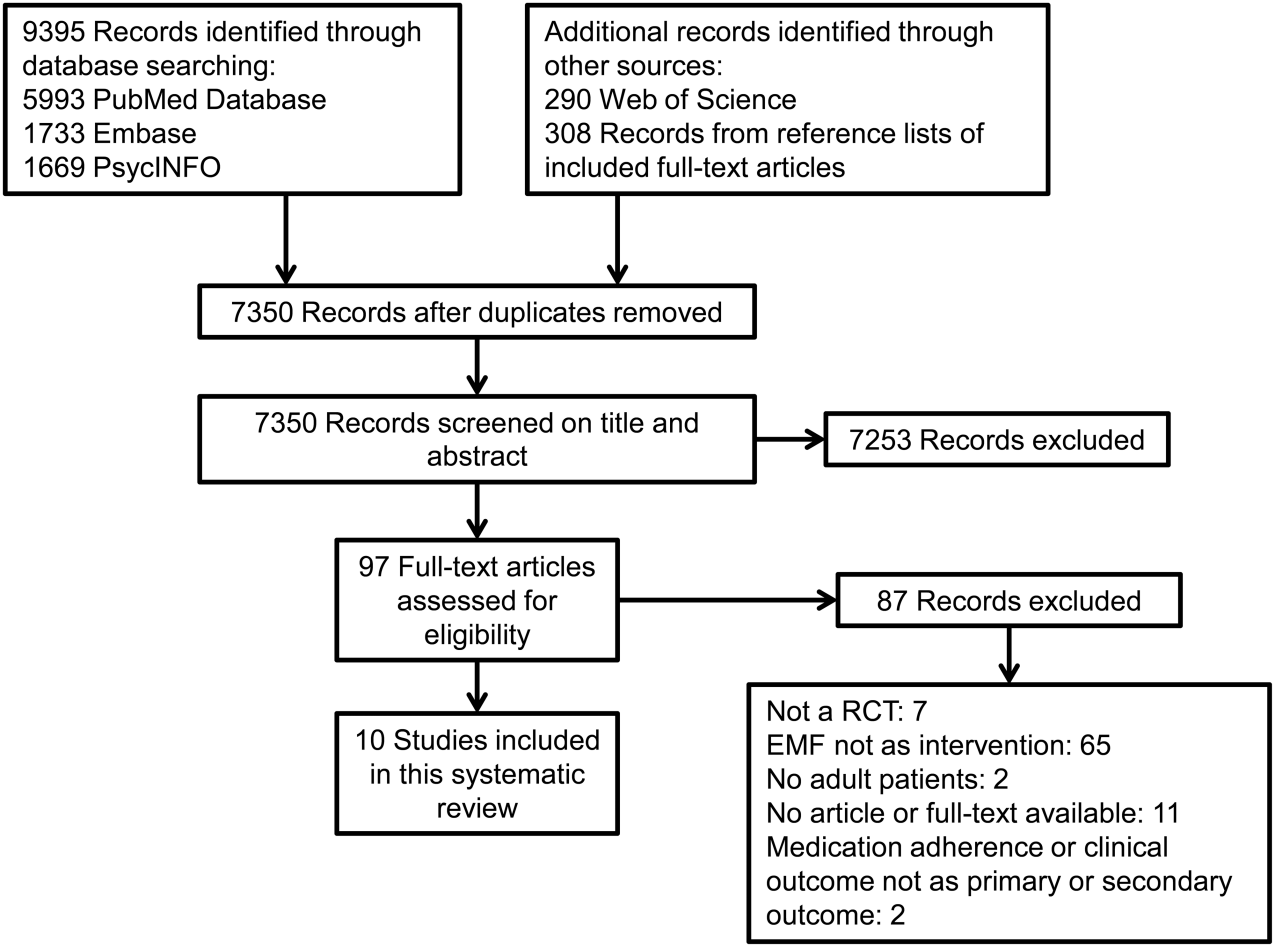
Flow diagram of the study selection process. This flow diagram represents the number of records identified, screened, included and excluded during the study selection process. A literature search was performed in Pubmed database, PsycINFO, EMBASE and Web of Science up until August 8^th^, 2017.

The literature search provided a total of 7,350 unique records that were screened for eligibility. After screening titles and abstracts 7,253 articles were excluded, which led to a total of 97 articles to screen as full-text. Of the 97 articles that were assessed for eligibility, ten articles had no abstract or full text available. An overview of the study selection process is shown in Fig 1. Ten RCTs met the inclusion criteria and were included in this systematic review. Reference lists of these included studies were checked, but resulted in no additional records that met the inclusion criteria.

### Study characteristics

An overview of the sample and intervention characteristics of the included studies is presented in Table 1.

**Table 1:** Study sample and intervention characteristics of the included studies.

	Study population characteristics				Intervention characteristics					
First author,year of publication	Indication	IG^a^ (n)	CG^b^ (n)	Loss to follow-up (%)	Device	Study duration (weeks)	Number of interventions	Feedback provider	Mean duration single intervention (min)	Description of the intervention (feedback)
Onyirimba, 2003 [25]	Asthma	30^c^		37	MDI Chronologs Model MC-311	10	4	Clinician	30–60	Computer printout (date and time)
										Discussion about actuation data at every follow-up visit after a period of monitoring (1 or 3 weeks, non-instant feedback)
										Direct clinician-to-patient feedback
										Non-judgmental discussion of results (constructive and positive) emphasising techniques and strategies to improve adherence
Schmitz, 2005 [26]	Smoking cessation	51	46	53	MEMS^®^ cap (without digital displays)	7	7	Clinical nurse	5–10	Graphic feedback after one week of monitoring (repeated)
										Feedback between patient and provider (non-instant feedback)
										Repeating instructions for improving adherence
										Check medication side effects
De Geest, 2006 [27]	Renal transplantation	6	12	28	Electronic device, not specified	13	4	Nurse	NA	Graphic and tabulated feedback (printouts) at the end of the month for three consecutive months
										Problem detection, proxy goal setting and regular targeted feedback
										Discussion about possible factors related to non-adherence, self-efficacy interventions
										Individualised intervention strategies (refreshment course, possible behavioural interventions and social support interventions that were based on an individualised assessment of reasons for non-adherence)
										One home visit, three follow-up calls by telephone
Mooney, 2007 [28]	Smoking cessation	27	28	56	MEMS^®^ cap (not specified)	6	6	Therapist	+10^d^	Graphic feedback based on individual cognitive behavioural therapy (CBT)
										Weekly feedback by a provider based on previous monitoring (non-instant feedback)
										Reviewing and clarifying the treatment regimen
										Problem-solving techniques to help the participant tailor the medication regime
										Identification of potential barriers and discussion about strategies to remove these barriers
										Encouraging patients to self-monitor pill consumption (daily diary)
										Evaluation self-monitoring at the next therapy session
Santschi, 2008 [29]	Hypertension	34	34	NA	MEMS^®^ cap (not specified)	52^e^	4^e^	Pharmacist	NA	Adherence report (printout)
										Discussion of the electronically recorded results over the past 2 months (non-instant feedback)
										In case of substantial drug omissions, GP and pharmacist explored ways to improve medication adherence with the patient.
Sabin, 2010 [30]	HIV positive	34	34	6	Med-ic^™^ pill bottle	26	6	Physician/ nurse	10–15	Graphic feedback (printout)
										Feedback by a provider based on previous month’s electronic drug monitor data (repeated, non-instant feedback)
										‘Flagged’ for counselling with a physician or nurse in case of adherence levels <95%
										Individually-focused discussion (no script)
										Questions focusing on missed or off-time doses, problems or challenges
De Bruin, 2010 [31]	HIV positive	66	67	13	MEMS^®^ cap (including a 24h LCD readout)	39	2 or more	HIV-nurse	NA	Graphic feedback
										Discussion of MEMS reports after a 3-month intervention period (non-instant feedback)
										Instant feedback by a MEMS-view-cap during the 2 to 4-month follow-up period
										‘Minimal intervention’: feedback of MEMS reports, reinforcement, and brief discussion about any difficulties that could be solved
										‘Full intervention’: increase knowledge, correct misconceptions when relevant, select a desired yet feasible adherence level including motivation for this choice, discussion about difference between actual and desired adherence status, identifying causes for non-adherence and tailored solutions for these problems, goal setting, option to self-monitor medication intake during the upcoming period to identify challenging situations and to develop solutions to cope with challenges.
Brath, 2013 [32]	Patients at risk for cardiovascular conditions (at least two out of three health risks: type 2 diabetes, hypertension, hypercholesterolaemia)	77 (crossover design)		31	Electronic medication blister (OtCM, DSM TCG B.V.)	52	Depends on individual adherence level	Study coordinator	NA	Adherence data were transmitted wirelessly by a NFC-enabled reader device (mobile phone)
										A web-based telehealth system analysed the received data
										Processed data were used to remind patients automatically via SMS messages
										Data were presented to the study physician numerically and graphically via browser-based user interface
										SMS reminders to remind patients to transmit data regularly and on time (instant feedback)
										Subjects with minor adherence (<70% of prescribed drugs taken) were contacted by a study coordinator once a week trying to increase adherence motivation (phone call, non-instant feedback)
Forni Ogna, 2013 [33]	Secondary hyperparathyroidism	24	26	18	MEMS^®^ Smartcap (including a 24h LCD readout)	26	3	Nephrologist	NA	Graphic feedback
										Feedback by a provider after 2 months of monitoring (repeated, non-instant feedback)
										Instant feedback by a 24h LCD readout MEMS cap
										Semi-structured motivational interviews
										Questions focusing on experience and complications with medication-intake
										Identification potential barriers and generation of strategies to overcome barriers
										Elaboration and evaluation individualised adherence plan
Dobbels, 2017 [34]	Heart, liver and lung transplant recipients	103	102	21	Helping Hand	26	2	Nurse with a Master in Nursing Science	NA	Graphic feedback (printouts of the 3-month monitoring period preceding the study visit) in a non-judgmental way delivered by the intervention giver (non-instant feedback)
										Patients with good implementation of medication regimen based on printouts of Helping Hand: social support (emotional), problem solving (including relapse prevention and coping planning)
										Patients with poor implementation of medication regimen based on printouts of Helping Hand: assessment of patient’s level of motivation using a 10-point importance and confidence ruler
										Instant feedback: the Helping Hand reminder system consists of a beeping signal at time of scheduled medication intake
										Instant feedback: the Helping Hand system provides visual signal feedback using a traffic light system (green, orange or red light)

^a^ number of included patients in the intervention group,

^b^ number of included patients in the control group,

^c^ allocation not mentioned,

^d^ Additional time required for the intervention, excluding regular time for a consult,

^e^ Depending on the results of office blood pressure (<140/90 mmHg) and adherence (≥80% taking adherence), the GP had the opportunity to continue or stop electronic monitoring. So, intervention period and follow-up period were variable. NA: not available

### Sample characteristics

The main differences between studies were sample size and length of follow-up. Also the condition of the study population in which the intervention was studied varied considerably (asthma, smoking cessation, renal, heart, liver and lung transplantation, hypertension, HIV, secondary hyperparathyroidism and patients at risk from cardiovascular conditions) [25–34]. However, the efficacy of EMF in HIV positive patients and smoking cessation was reported by two studies for both conditions [26;28;30;31]. Most studies used MEMS as an electronic device to monitor medication adherence, except for Onyirimba et al., Brath et al. and Dobbels et al., in which metered-dose inhalers, electronic medication blisters and the Helping Hand tool with a traffic light system (green, orange and red light) were used, respectively [25,32,34]. De Geest et al. also used an electronic device, but this device was not further specified [27]. Forni Ogna et al. and De Bruin et al. used MEMS devices with 24h LCD read-outs to provide patients with feedback [31,33]. Although Brath et al. used electronic medication blisters (non-instant feedback) instead of MEMS view-caps, patients were also indirectly provided with instant feedback due to SMS reminders to transmit data regularly and on time [32]. Instant feedback was also provided with the Helping Hand reminder system which consists of a beeping signal at time of scheduled medication intake [34]. Variability in costs of study medication and socio-economic status of participants were also noted.

Six studies had dose adherence as the primary outcome, while only three studies reported both full- and dose adherence. Santschi et al. reported median adherence levels, but not mean adherence levels on dose- and full adherence [29]. The effect of EMF on clinical outcomes was reported by seven studies (three studies as primary outcome and four studies as secondary outcome). Brath et al. only reported the effect of EMF on clinical outcomes at the beginning and at the end of the crossover phase, thereby, representing inappropriate data for including these data in our overview [32]. An overview of the effect of EMF on medication adherence and/or clinical outcomes is presented in Table 2.

**Table 2:** Effect of EMF on dose adherence, full adherence and clinical outcomes.

First author, year of publication	Indication	Intervention period	Follow-up period	Dose adherence (%)			Full adherence (%)			Clinical outcome				Overall score	
		Weeks	Weeks	I(SE)	C (SE)	P-value	I(SE)	C (SE)	P-value	Outcome measurement	I (SD)	C (SD)	P-value	Medication adherence	Clinical outcomes
Onyirimba, 2003 [25]	Asthma	3	7	75 (6)	26 (7)	S	NA	NA	NA	FEV1 (L)	+0.04 (0.11)	+0.16 (11)	0.44	+	NA
										Mean weekly 24-h albuterol use	1.51 (NA)	2.09 (NA)	NA		
										Mean weekly nightly (01.00–05.00 AM) albuterol use	0.07 (NA)	0.13 (NA)	NA		
										Asthma Quality of Life Questionnaire	1.13 ± 0.31	0.76± 0.33	<0.05		
Schmitz, 2005 [26]	Smoking cessation	7	NA	64 (6)	36 (5)	<0.05	48 (5)	19 (6)	<0,05	NA	NA	NA	NA	+	NA
De Geest^a^, 2006 [27]	Renal transplantation	13	26	97.0 (2.1)	93.4 (9.6)	0.58	NA	NA	NA	NA	NA	NA	NA	-	NA
Mooney, 2007 [28]	Smoking cessation	6	NA	62 (8)	35 (7)	S	40 (9)	18 (7)	S	Abstinence^b^	r(35)_dose compliance_ = 0.38		NS	+	-
											r(35)_full compliance_ = 0.40		NS		
Santschi^c^, 2008 [29]	Hypertension	NA^d^	NA^d^	NA	NA	NA	NA	NA	NA	Blood pressure: systolic/ diastolic (mmHg)^e^	149 (3.7)/ 87 (1.9)	154 (2.3)/ 84.5 (2.6)	0.21/0.40	NA	-
Sabin, 2010 [30]	HIV	26	NA	NA	NA	NA	96.5 (4.8^f^)	84.5 (21^f^)	0.003	CD4 count at month 12 (cells/μL)	401 (256^f^)	357 (157^f^)	0.410	+	-
										Change in CD4 count (cells/μL) month 6 to 12	+90.0 (171.6^f^)	-8,8 (152.6^f^)	0.020		
										HIV RNA <400 copies/ml at month 12, n (%)	27 (87.1)	31 (93.9)	0.3518		
De Bruin, 2010 [31]	HIV	12	16	96	90	<0,001	82	65	<0.001	Detectable plasma HIV-RNA (dichotomized, >, 50 copies/ml), n (%)	6 (9.4)	13 (20.6)	NA	+	NA
										Changes in plasma HIV-RNA (copies/ml)	OR [95%CI] = 2.96 [1,00–8.74]		<0.05		
Brath, 2013 [32]	Patients with a defined risk for cardiovascular conditions	20	8	NA	NA	NA	NA	NA	NA	Fasting plasma glucose (mg dl^-1^)	NA		NA	NA	NA
										HbA1c (%)	NA		NA		
										Body weight (kg)\	NA		NA		
										Blood pressure sys/dia (mmHg)	NA		NA		
										Total cholesterol (mg dl^-1^	NA		NA		
										LDL cholesterol (mg dl^-1^)	NA		NA		
										HDL cholesterol (mg dl^-1^)	NA		NA		
Forni Ogna, 2013 [33]	Secondary Hyper-parathyroidism	26	13	94.7 (NA)	88.2 (NA)	NA	NA	NA	NA	Median absolute intact parathyroid hormone (iPTH) at month 6 (ng/L (interquartile range: IQR))	339(236, 529)	436 (288, 682)	0.05	NA	+
										Median iPTH change at month 6 (ng/L, IQR)	-94.3 (-282.6, -27.7)	+113.6 (-26.2, 145.1)	0.009		
										Median iPTH change after 3 months follow-up, month 9 (ng/L, IQR)	+50.0 (-85.8, +304.1)	+0.16 (-163.3, 197.1)	NA		
Dobbels, 2017 [34]	Heart, liver and lung transplant recipients	26	26	NA	NA	NA	92.1 (NA)	72.1 (NA)	<0.0001	5-year clinical event-free survival	82.5%	72.5%	0.18	+	-
											RR [95%CI] = 0.64 [0.38–1.08]				

^a^ results show a decrease in non-adherence in both groups; difference did not reach statistical significance,

^b^ results for week 6 (no data for week 10 available); point-biserial correlations on the pooled sample,

^c^ standard error of the mean (SEM),

^d^ Depending on the results of office blood pressure (<140/90 mmHg) and adherence (≥80% taking adherence) measured with electronic device, the GP had the opportunity to continue or stop electronic monitoring. So intervention period and follow-up period are variable.

^e^ systolic blood pressure (mmHg)/ diastolic blood pressure (mmHg). This ratio also applies to the P-value (p-value systolic blood pressure/ p-value diastolic blood pressure)

^f^ standard deviation (SD).

S: statistical significant, but p-value unknown, NS: statistical not significant, NA: not available, I: intervention arm, C: control arm, Dose adherence: number of doses taken divided by the number of doses prescribed, Full adherence: dose adherence in the correct time schedule.

### Feedback characteristics

The feedback varied between studies regarding the duration of a single feedback session, feedback provider, type of electronic device (metered dose inhalers, electronic medication blisters, Helping Hand, MEMS with or without 24h LCD readout), total intervention duration, the number of interventions in the specified study period and the components of the feedback. Most studies used a graphic form to confront patients with their medication-intake behaviour, and described face-to-face feedback [25–34]. However, De Geest et al. and Brath et al. used telephone calls to give feedback in the follow-up period [27,32]. Most studies did not describe the precise method of giving feedback to patients, as conversations did not follow a structured way. Only Forni Ogna et al. and Dobbels et al. used a semi-structured motivational interviewing method to discuss the results with patients in the intervention arm [33,34]. The monitoring phase prior to delivering feedback also varied between studies [25–34]. In the study of Schmitz et al. patients received graphic feedback after a week of monitoring, whereas in the study of de Bruin et al. the feedback was delivered after three months of monitoring [26,31]. The following issues were highlighted most during a feedback session: identification of potential barriers, strategies to remove barriers, exploration of strategies to improve adherence, verification of medication side-effects and evaluation of the previously elaborated adherence plan at the next visit.

### Quality assessment

Of the ten studies included in this systematic review, four studies met the criteria for high-quality studies, as described in the Methods section. Studies classified as low-quality studies were studies with shortcomings on statistics, treatment allocation, protocol adherence, and description of withdrawal or drop-out rate. An overview of the performed quality assessment, including scores for critical and less critical criteria, is represented in Table 3.

**Table 3:** Quality assessment of individual studies based on critical and less critical criteria.

First author,year of publication	Critical criterion (%)	Less critical criterion (%)	Quality assessment individual study
Onyirimba, 2003 [25]	38	63	Low
Schmitz, 2005 [26]	63	47	Low
De Geest, 2006 [27]	50	67	Low
Mooney, 2007 [28]	50	27	Low
Santschi, 2008 [29]	75	67	High
Sabin, 2010 [30]	88	73	High
Forni Ogna, 2013 [33]	63	60	Low
De Bruin, 2010 [31]	88	63	High
Brath, 2013 [32]	25	31	Low
Dobbels, 2017 [34]	100	63	High

### Summary of the results: Medication adherence

Ten randomised controlled trials studied the effect of EMF on medication adherence. Of these ten studies, Brath et al. used a crossover design without a ‘wash-out period’ which made these results difficult to interpret, since the intervention effect from the monitoring phase could still exist in the control phase [32]. Santschi et al. and Brath et al. reported median adherence levels instead of mean adherence levels compared with other included studies [29,32]. In the study of Santschi et al., only the intervention group was monitored with MEMS devices [29]. An overview of the effect of EMF on mean dose adherence and full adherence levels is presented in Table 2.

The effect of EMF on mean dose adherence levels after the intervention period was studied in six randomised controlled trials. Of these six studies, four studies reported a significant positive effect of EMF on mean dose adherence levels. De Geest et al. reported a non-significant effect between both groups and Forni Ogna et al. did not describe the level of significance between the intervention and control arm [27,33]. Considering the number of included studies that described a significant positive effect (66.7%, four out of six included studies) of EMF on dose adherence, the overall effect of EMF on mean dose adherence levels was positive. A significant positive effect of EMF was also reported on mean full adherence levels. All of the five studies (100%) that described the effect of EMF on mean full adherence levels reported a significant positive effect.

Overall, the studies included showed a positive significant effect of EMF on dose (66.7%) and full adherence levels (100%), since at least 66.7% of the reported adherence outcomes at the moment of the last intervention or the first results after the intervention has been stopped were significant.

The high degree of heterogeneity in intervention characteristics and sample characteristics prevented pooling data from individual studies.

The strength of the body of evidence for the efficacy of EMF on medication adherence was very low (for a detailed description of the quality rating process, see S3 Table).

### Summary of the results: Clinical outcomes

Seven randomised controlled trials studied the effect of EMF on clinical outcomes [25,28–33]. Of these seven studies, four studies (HIV progression, secondary hyperparathyroidism and asthma) reported a significant effect of EMF on at least one clinical outcome [25,30,31,33]. An overview of the effect on clinical outcomes due to EMF is presented in Table 2. Four studies reported positive as well as negative findings on clinical outcome measures [25,30,31,33]. These findings also varied over time, as described by Forni Ogna et al., which reported a significant effect on intact parathyroid hormone level at month six, but no significant effect at month nine [33].

Although some studies reported a significant effect on at least one clinical outcome due to EMF, only one out of the six studies included had a positive overall score (at least 66.7% of the reported clinical outcomes at the moment of the last intervention or the first results after the intervention had been stopped were significant, as presented in Table 2, last column). The high degree of clinical heterogeneity across studies prevented pooling of data from individual studies.

## Discussion

### Summary of evidence

This systematic review demonstrates that EMF tends to have a positive effect on medication adherence (mean dose- and full adherence levels) and an inconclusive effect on clinical outcomes. The majority of the studies included were characterised by a low methodological quality and a high degree of clinical heterogeneity (i.e. patient, disease and study characteristics,). In addition, the diversity in intervention characteristics (i.e. no or semi-structured interviews/feedback, different feedback providers, different electronic devices, different frequencies of the intervention) might have contributed to the lack of evidence for the efficacy of this intervention. These study characteristics prevented pooling of data on adherence and clinical outcomes. However, the overall positive effect of EMF on medication adherence supports the hypothesis that EMF might be a promising strategy to enhance both dose- and full adherence.

### Literature

To our knowledge, this is the first systematic review that summarises the single effect of EMF on medication adherence and clinical outcomes. Our results confirm the findings of Demonceau et al. (a systematic review including interfering co-interventions and, therefore, not examining the single effect of EMF) that EMF-feedback is a potentially effective approach to enhance patient’s adherence to medication [7].

Although improving clinical outcomes and patient’s quality of life are still the ultimate goals of adherence-improving interventions, clinical outcomes and/or quality of life are often not chosen as primary outcomes in adherence-improving studies [3]. We did not find evidence for the efficacy of EMF on clinical outcomes. This lack of evidence is most likely due to clinical heterogeneity across the studies included and small sample sizes.

There is also a time-to-event component in longitudinal adherence-improving studies. Although the effect of EMF on medication adherence is often expressed in dose- and full adherence, persistence and non-persistence may occur during this monitoring period. Measuring dose- and full adherence over a long period to provide feedback to patients might be inappropriate. However, these methodological insights in adherence-improving studies are slowly implemented in intervention studies. Therefore, we decided to study the effect of EMF on dose- and full adherence as primary outcomes in this review.

In contrast to the Cochrane review of Nieuwlaat et al., we did not exclude studies with ≤80% follow-up [3]. Our review included studies with a loss to follow-up range that varied from 6 to 56% (three studies would meet the inclusion criteria used in the Cochrane review). In this regard, our review provides a more realistic appraisal of the effect of EMF as a high degree of loss to follow-up often exists in adherence-improving studies and might also be the case in usual care.

### Strengths and limitations

The most important limitation of this review is the lack of a worldwide definition regarding the content of electronic monitoring feedback, as no criteria for the minimum requirements of the intervention are described. This broad definition is considered to be the main reason for the high degree of heterogeneity in intervention characteristics of the included studies, making it either very complex to compare the efficacy of EMF on adherence in the different studies, and also reducing the possibility to assess the impact of the different intervention characteristics on the efficacy of EMF. We considered it not appropriate to perform additional analyses to evaluate the influence of heterogeneity in both intervention and study characteristics (including the quality of studies) due to the small number of studies included in this review, and therefore, a possible incorrect interpretation of the results.

In general, there is always a chance of publication bias, as positive studies are more likely to be published compared to negative studies. This might lead to an overestimation of the actual effect.

Also the use of electronic devices in the control arm (without the non-instant feedback component) in adherence-improving studies might increase patient’s awareness of adequate medication-intake and consequently improve medication adherence. This awareness might decrease the contrast between the intervention and control arm, and thus effect sizes between groups. Furthermore, the type of electronic device might have influenced adherence levels, as MEMS with LCD displays (or the Helping Hand tool) provide instant feedback, whereas MEMS devices without LED displays provide no instant feedback. Feedback by a provider instead of a MEMS device has the advantage that both intentional and unintentional non-adherent behaviour can be targeted. Nevertheless, feedback by a provider after a (long) monitoring period might be less effective than instant feedback by a MEMS device in case of unintentional non-adherent behaviour. In addition, patients participating in a randomised clinical trial, irrespective of the allocated study arm and the use of an electronic device, might influence the results as usual care in a RCT is not usual care by definition.

At-home assistance is another potential concern of adherence-improving studies as adherence levels in both arms may be overestimated. Nearly all published adherence-improving studies do not evaluate or describe support from significant others (e.g. spouses and/or homecare). Therefore, there is often a lack of ability to control for support in the home context. However, support of significant others can also be used as an intervention component to improve medication adherence. For instance, in the study of Wu et al. the support of significant others was an essential part of the intervention [15]. Based on these general limitations of adherence-improving studies, individual studies are often characterized by a low methodological quality.

For the quality assessment of each study included in our review, consensus was achieved to differentiate the quality criteria in critical and less critical criteria. As all involved researchers agreed in a consensus meeting that an unequivocal majority of the critical quality criteria should be positive, a cut-off level of 75% was set for the critical criteria in order to assign a study as high-quality study. However, if a cut-off level of 50% for critical criteria had been agreed, six of the ten studies would have been assessed as high-quality studies. However, despite this, the strength of the body of evidence assessed with the GRADE approach did not change and remained very low.

### Future research

This review has demonstrated that EMF might be a promising strategy to enhance dose- and full adherence, although high-quality, sufficiently powered studies are required in future research. As a significant effect of EMF on clinical outcomes cannot yet be excluded, future research with optimal study methodologies is required.

Future high-quality research should firstly focus on the short- and long-term efficacy of EMF on medication adherence. Alongside efficacy, other aspects, such as the effect of the intervention giver, the duration of the intervention, and the patient follow-up should be assessed. The latter is essential as non-adherence to medication is a dynamic process and patients might become non-adherent when the intervention is not continued. Finally, when a significant effect of EMF on medication adherence has been shown in multiple high-quality studies, further research should focus on the efficacy of EMF on clinical outcomes and the implementation of EMF in clinical practice.

## Conclusions

This systematic review suggests that EMF might be a promising intervention in improving medication adherence. However, the efficacy of EMF on clinical outcomes is inconclusive. Considering the positive effect of EMF on medication adherence (mean dose- and full adherence levels) and the inconclusive results on clinical outcomes, evidence for the efficacy of EMF on both outcomes is limited. As our results are mainly based on low-quality studies, future research with high-quality studies is required to examine if EMF has significant effect on medication adherence. Only when a significant effect of EMF on medication adherence has been shown, studies should focus on the efficacy of EMF on clinical outcomes. Implementing EMF in clinical practice is not yet recommended.

## Supporting information

S1 Checklist PRISMA checklist. (DOCX)

S1 Appendix Search strategy. (DOCX)

S1 Table Quality assessment methods. (DOCX)

S2 Table Methods GRADE approach. (DOCX)

S3 Table Results GRADE approach. (DOCX)
