# Supplementary material for: The effect of electronic monitoring feedback on medication adherence and clinical outcomes: A systematic review
Source: PLoS One. 2017 Oct 9;12(10):e0185453. doi: 10.1371/journal.pone.0185453 (PMC5633170; doi:10.1371/journal.pone.0185453)
Supplement: S1 Appendix — (DOCX) [file pone.0185453.s002.docx]

## S1 Appendix. Search strategy

### Search strategy Pubmed

1. (medication adherence[MH]) OR (medication compliance[TW] OR compliance [tw] OR adherance [tw] OR adherence [tw] OR medication non-compliance[TW] OR medication non compliance[TW] OR medication noncompliance[TW] OR medication adherence[TW] OR medication non-adherence[TW] OR medication non adherence[TW] OR medication nonadherence[TW] OR medication adherance[TW] OR medication non-adherance[TW] OR medication non adherance[TW] OR medication nonadherance[TW] OR medication persistence[TW] OR medication non-persistence[TW] OR medication non persistence[TW] OR medication nonpersistence[TW] OR medication persistance[TW] OR medication non-persistance[TW] OR medication non persistance[TW] OR medication nonpersistance[TW] OR medicine compliance[TW] OR medicine non-compliance[TW] OR medicine non compliance[TW] OR medicine noncompliance[TW] OR medicine adherence[TW] OR medicine non-adherence[TW] OR medicine non adherence[TW] OR medicine nonadherence[TW] OR medicine adherance[TW] OR medicine non-adherance[TW] OR medicine non adherance[TW] OR medicine nonadherance[TW] OR medicine persistence[TW] OR medicine non-persistence[TW] OR medicine non persistence[TW] OR medicine nonpersistence[TW] OR medicine persistance[TW] OR medicine non-persistance[TW] OR medicine non persistance[TW] OR medicine nonpersistance[TW] OR medical compliance[TW] OR medical non-compliance[TW] OR medical non compliance[TW] OR medical noncompliance[TW] OR medical adherence[TW] OR medical non-adherence[TW] OR medical non adherence[TW] OR medical nonadherence[TW] OR medical adherance[TW] OR medical non-adherance[TW] OR medical non adherance[TW] OR medical nonadherance[TW] OR medical persistence[TW] OR medical non-persistence[TW] OR medical non persistence[TW] OR medical nonpersistence[TW] OR medical persistance[TW] OR medical non-persistance[TW] OR medical non persistance[TW] OR medical nonpersistance[TW] OR drug compliance[TW] OR drug non-compliance[TW] OR drug non compliance[TW] OR drug noncompliance[TW] OR drug adherence[TW] OR drug non-adherence[TW] OR drug non adherence[TW] OR drug nonadherence[TW] OR drug adherance[TW] OR drug non-adherance[TW] OR drug non adherance[TW] OR drug nonadherance[TW] OR drug persistence[TW] OR drug non-persistence[TW] OR drug non persistence[TW] OR drug nonpersistence[TW] OR drug persistance[TW] OR drug non-persistance[TW] OR drug non persistance[TW] OR drug nonpersistance[TW] OR drugs compliance[TW] OR drugs non-compliance[TW] OR drugs non compliance[TW] OR drugs noncompliance[TW] OR drugs adherence[TW] OR drugs non-adherence[TW] OR drugs non adherence[TW] OR drugs nonadherence[TW] OR drugs adherance[TW] OR drugs non-adherance[TW] OR drugs non adherance[TW] OR drugs nonadherance[TW] OR drugs persistence[TW] OR drugs non-persistence[TW] OR drugs non persistence[TW] OR drugs nonpersistence[TW] OR drugs persistance[TW] OR drugs non-persistance[TW] OR drugs non persistance[TW] OR drugs nonpersistance[TW])
2. ((((((((randomized controlled trial[Publication Type]) OR controlled clinical trial[Publication Type]) OR randomized[Title/Abstract]) OR placebo[Title/Abstract]) OR drug therapy[MeSH Subheading]) OR randomly[Title/Abstract]) OR trial[Title/Abstract]) OR groups[Title/Abstract])
3. (electronic [tw] OR electronically [tw] OR MEMS [tw] OR medication event monitoring system [tw] OR EDM [tw] OR electronic dose monitoring [tw] OR MGMM [tw] OR Measurement-Guided Medication Management [tw] OR EMF [tw] OR EM-feedback [tw] OR electronically monitored adherence feedback [tw] OR electronic-monitoring adherence feedback [tw] OR electronically monitored feedback [tw] OR electronic medication-event monitoring [tw] OR electronically-compiled dosing [tw] OR electronic medication-event methods [tw] OR smartinhaler [tw] OR Doser CT [tw] OR RemindRX [tw] OR dosing aid [tw] OR diskus adherence logger [tw] OR DAL [tw] OR metered dose inhaler [tw] OR MDI [tw] OR Med-Ic [tw] OR SIMPill [tw])
4. 1 AND 2 AND 3

### Search strategy EMBASE

1. exp medication compliance/
2. exp patient compliance/
3. exp treatment refusal/
4. 1 or 2 or 3
5. (medication compliance or medication non-compliance or medication non compliance or medication noncompliance or medication adherence or medication non-adherence or medication non adherence or medication nonadherence or medication adherance or medication non-adherance or medication non adherance or medication nonadherance or medication persistence or medication non-persistence or medication non persistence or medication nonpersistence or medication persistance or medication non-persistance or medication non persistance or medication nonpersistance or medicine compliance or medicine non-compliance or medicine non compliance or medicine noncompliance or medicine adherence or medicine non-adherence or medicine non adherence or medicine nonadherence or medicine adherance or medicine non-adherance or medicine non adherance or medicine nonadherance or medicine persistence or medicine non-persistence or medicine non persistence or medicine nonpersistence or medicine persistance or medicine non-persistance or medicine non persistance or medicine nonpersistance or medical compliance or medical non-compliance or medical non compliance or medical noncompliance or medical adherence or medical non-adherence or medical non adherence or medical nonadherence or medical adherance or medical non-adherance or medical non adherance or medical nonadherance or medical persistence or medical non-persistence or medical non persistence or medical nonpersistence or medical persistance or medical non-persistance or medical non persistance or medical nonpersistance or drug compliance or drug non-compliance or drug non compliance or drug noncompliance or drug adherence or drug non-adherence or drug non adherence or drug nonadherence or drug adherance or drug non-adherance or drug non adherance or drug nonadherance or drug persistence or drug non-persistence or drug non persistence or drug nonpersistence or drug persistance or drug non-persistance or drug non persistance or drug nonpersistance or drugs compliance or drugs non-compliance or drugs non compliance or drugs noncompliance or drugs adherence or drugs non-adherence or drugs non adherence or drugs nonadherence or drugs adherance or drugs non-adherance or drugs non adherance or drugs nonadherance or drugs persistence or drugs non-persistence or drugs non persistence or drugs nonpersistence or drugs persistance or drugs non-persistance or drugs non persistance or drugs nonpersistance).af
6. 4 or 5
7. (randomized controlled trial* or randomised controlled trial* or clinical trial* or controlled clinical trial*).mp.
8. exp microelectromechanical system/
9. (electronic or electronically or electronic monitoring or electronic monitoring feedback or MEMS or medication event monitoring system or EDM or electronic dose monitoring or MGMM or Measurement-Guided Medication Management or EMF or EM-feedback or electronically monitored adherence feedback or electronic-monitoring adherence feedback or electronically monitored feedback or electronic medication-event monitoring or electronically-compiled dosing or electronic medication-event methods or smartinhaler or Doser CT or RemindRX or dosing aid or diskus adherence logger or DAL or metered dose inhaler or MDI OR Med-Ic or SIMPill).af.
10. 8 or 9
11. 6 AND 7 AND 10

### Search strategy PsycINFO

- 1. exp Treatment compliance/
  2. (medication compliance or medication non-compliance or medication non compliance or medication noncompliance or medication adherence or medication non-adherence or medication non adherence or medication nonadherence or medication adherance or medication non-adherance or medication non adherance or medication nonadherance or medication persistence or medication non-persistence or medication non persistence or medication nonpersistence or medication persistance or medication non-persistance or medication non persistance or medication nonpersistance or medicine compliance or medicine non-compliance or medicine non compliance or medicine noncompliance or medicine adherence or medicine non-adherence or medicine non adherence or medicine nonadherence or medicine adherance or medicine non-adherance or medicine non adherance or medicine nonadherance or medicine persistence or medicine non-persistence or medicine non persistence or medicine nonpersistence or medicine persistance or medicine non-persistance or medicine non persistance or medicine nonpersistance or medical compliance or medical non-compliance or medical non compliance or medical noncompliance or medical adherence or medical non-adherence or medical non adherence or medical nonadherence or medical adherance or medical non-adherance or medical non adherance or medical nonadherance or medical persistence or medical non-persistence or medical non persistence or medical nonpersistence or medical persistance or medical non-persistance or medical non persistance or medical nonpersistance or drug compliance or drug non-compliance or drug non compliance or drug noncompliance or drug adherence or drug non-adherence or drug non adherence or drug nonadherence or drug adherance or drug non-adherance or drug non adherance or drug nonadherance or drug persistence or drug non-persistence or drug non persistence or drug nonpersistence or drug persistance or drug non-persistance or drug non persistance or drug nonpersistance or drugs compliance or drugs non-compliance or drugs non compliance or drugs noncompliance or drugs adherence or drugs non-adherence or drugs non adherence or drugs nonadherence or drugs adherance or drugs non-adherance or drugs non adherance or drugs nonadherance or drugs persistence or drugs non-persistence or drugs non persistence or drugs nonpersistence or drugs persistance or drugs non-persistance or drugs non persistance or drugs nonpersistance).af.
  3. 1 or 2
  4. (randomized controlled trial* or randomised controlled trial* or clinical trial* or controlled clinical trial*).mp.
  5. (electronic or electronically or electronic monitoring or electronic monitoring feedback or MEMS or medication event monitoring system or EDM or electronic dose monitoring or MGMM or Measurement-Guided Medication Management or EMF or EM-feedback or electronically monitored adherence feedback or electronic-monitoring adherence feedback or electronically monitored feedback or electronic medication-event monitoring or electronically-compiled dosing or electronic medication-event methods or smartinhaler or Doser CT or RemindRX or dosing aid or diskus adherence logger or DAL or metered dose inhaler or MDI OR Med-Ic or SIMPill).af.
  6. 3 and 4 and 5
