# Supplementary material for: The effect of electronic monitoring feedback on medication adherence and clinical outcomes: A systematic review
Source: PLoS One. 2017 Oct 9;12(10):e0185453. doi: 10.1371/journal.pone.0185453 (PMC5633170; doi:10.1371/journal.pone.0185453)
Supplement: S1 Table — (DOCX) [file pone.0185453.s003.docx]

## S1 Table. Quality assessment methods

|  | | | **Critical criteria** | **Less critical criteria** | |
| --- | --- | --- | --- | --- | --- |
| **Study design** | | |  |  | |
| Setting | | |  | x | |
| **Patient selection** | | |  |  | |
| Eligibility criteria specified | x |  | |  |  |
| Methods of inclusion/selection |  | x | |  |  |
| Treatment allocation: randomization |  | x | |  |  |
| Treatment allocation: concealed |  | x | |  |  |
| Similarity of groups at baseline | x |  | |  |  |
| Report number of individuals at each stage of study |  | x | |  |  |
| Reasons for non-participation |  | x | |  |  |
| **Interventions** | | |  |  | |
| Explicit description of exposure | x |  | |  |  |
| Blinding of care provider |  | x | |  |  |
| Avoidance of co-interventions^a^ | x |  | |  |  |
| Acceptable compliance |  | x | |  |  |
| Blinding of patient |  | x | |  |  |
| **Outcome measurement** | | |  |  | |
| Blinding of outcome assessor |  | x | |  |  |
| Relevance of outcome measures | x |  | |  |  |
| Description of adverse effects^b^ |  | x | |  |  |
| Description of withdrawal/drop-out rate |  | x | |  |  |
| Acceptable drop-out rate if study sample size ≤ 50 participants: positive, when total number of participants lost to follow-up was < 20% at follow-up ≥ 3 months. If study sample size > 50 participants: positive, when total number of participants lost to follow-up was < 33% at follow-up ≥ 3 months. | x |  | |  |  |
| Timing follow-up measurement correct |  | x | |  |  |
| Timing of outcome measurement in both groups comparable |  | x | |  |  |
| **Statistics** | | |  |  | |
| Power analysis | x |  | |  |  |
| Intention-to-treat analysis included |  | x | |  |  |
| Presentation of point estimated and measures of variability | x |  | |  |  |
| Loss to follow up |  | x | |  |  |
| ^a^ studies with radical co-interventions were excluded; for instance articles scored negative if the usual care comprises enhanced usual care (and where both groups received this enhanced usual care)  ^b^ It is assumed that EMF as intervention will not lead to adverse effects. For this reason description of adverse effects as desirable criterion is excluded. | | | |  |  |
