# Supplementary material for: The effect of electronic monitoring feedback on medication adherence and clinical outcomes: A systematic review
Source: PLoS One. 2017 Oct 9;12(10):e0185453. doi: 10.1371/journal.pone.0185453 (PMC5633170; doi:10.1371/journal.pone.0185453)
Supplement: S2 Table — (DOCX) [file pone.0185453.s004.docx]

| **GRADE-domain** | **Instrument used** | **Quality indicator** | **Cut of quality indicator for each individual study** | **Criteria for downgrading evidence** |
| --- | --- | --- | --- | --- |
| Risk of bias | Quality assessment of individual studies | Adequate description of patient selection, allocation, blinding and loss to follow up in each study | > 75% of the essential criteria and >50% of the desirable criteria are adequately described; otherwise negative score | -1: >25% of the patients are included in a study with considerable risk of bias |
| Inconsistency | Forest plot | Extent of overlap CI, visual inspection |  | -1: CI little or none overlap |
|  | Test for heterogeneity | I^2^ |  | -1: I^2^ >0.5 |
|  |  | p-value |  | -1: P <0,10 |
| Imprecision | Quality assessment of individual studies | Power analysis described and included patients according to power analysis | No power analysis described | -1: >25% of the studies has a negative score |
|  |  |  | Number of patients included smaller than number of patients calculated |  |
|  | Optimal information size | Optimal information size calculated compared to sum of the patients in the individual studies |  | -1: optimal information size > sum of the patients in individual studies |
| Indirectness | Quality assessment individual studies | Description population, intervention, outcome measures | > 75% of the scoring items are adequately described, otherwise negative score | -1: >25% of studies has a negative score |
|  |  | Comparison within or between studies |  | -1: indirect comparison |
| Publication bias | Quality assessment individual studies | Funding | Industry sponsoring described | -1: >75% of studies sponsored |
|  | Funnel plot ^a^ | Asymmetry of the plot |  | -1: asymmetrically distributed |

## S2 Table. Methods GRADE approach
