# Supplementary material for: The effect of electronic monitoring feedback on medication adherence and clinical outcomes: A systematic review
Source: PLoS One. 2017 Oct 9;12(10):e0185453. doi: 10.1371/journal.pone.0185453 (PMC5633170; doi:10.1371/journal.pone.0185453)
Supplement: S3 Table — (DOCX) [file pone.0185453.s005.docx]

| **Patient outcome** | **Medication adherence** | | | | | | | | | |  |  |  |
| --- | --- | --- | --- | --- | --- | --- | --- | --- | --- | --- | --- | --- | --- |
|  | **Study characteristics** | | | **Criteria GRADE approach: downgrading quality of evidence** | | | | | | | **Criteria GRADE approach: upgrading quality of evidence** | | |
|  | **Disease** | **Number of patients** | **Type of study** | **Risk of bias** | **Inconsistency** | | **Indirectness** | **Imprecision** | **Publication bias** | | **Large magnitude of the studied effect** | **Dose-response gradient** | **All plausible confounding would reduce the demonstrated effect or suggest a spurious effect when results show no effect** |
| **Mooney et al. (2007)** | Smoking cessation | 55 | RCT | - | No forest plot due to small number of studies and a high degree of heterogeneity in intervention characteristics and sample characteristics. | | - | - | No funding | No funnel plot due to small number of studies | - | - | - |
| **Schmitz et al. (2005)** | Smoking cessation | 97 | RCT | - |  |  | - | - |  |  | - | - | - |
| **Onyirimba et al. (2010)** | Asthma | 30 | RCT | - |  |  | - | - |  |  | - | - | - |
| **Sabin et al. (2010)** | HIV | 68 | RCT | + |  |  | + | + |  |  | - | - | - |
| **De Bruin et al. (2010)** | HIV | 133 | RCT | + |  |  | - | - |  |  | - | - | - |
| **Brath et al. (2013)** | Patients with a defined risk for cardio-vascular conditions | 77 | RCT | - |  |  | - | - |  |  | - | - | - |
| **Forni Ogna et al. (2013)** | Secondary hyperparathyroidism | 50 | RCT | - |  |  | - | - |  |  | - | - | - |
| **Dobbels et al. (2017)** | Heart, liver and lung transplant recipients | 205 | RCT | + |  |  | + | + |  |  | - | - | - |
| **Conclusion** | Various conditions | Total of 715 patients | All studies were RCTs | 56.8% of the patients is included in a study with considerable risk of bias | 0 | 0 | Comparison within studies | More than 25% of the studies has a negative score | 0 | 0 | 0 | 0 | 0 |
| **GRADE score** |  |  | 3 | -1 | 0 | | -1 | -1 | 0 | | 0 | 0 | 0 |
| **Quality of evidence** | **The quality of evidence for the effect of EMF on medication adherence is very low (GRADE score 0)** | | | | | | | | | | | | |

## S3 Table. Results GRADE approach
